# The clinical utility of ventilation–perfusion scintigraphy in the classification of pulmonary hypertension

**DOI:** 10.1007/s11604-025-01822-5

**Published:** 2025-06-27

**Authors:** Yoichi Otomi, Hideki Otsuka, Ryosuke Kasai, Jo Morishita, Minaho Miyake, Ko Minote, Kohei Furutani, Mana Shimomura, Yushi Kamei, Hiroto Kasai, Takayoshi Shinya, Masafumi Harada

**Affiliations:** 1 Department of Radiology, Tokushima University Hospital, 2-50-1 Kuramoto-Cho, Tokushima, 770-8503 Japan; 2 Department of Medical Imaging/Nuclear Medicine, Tokushima University Graduate School of Biomedical Sciences, Tokushima, Japan; 3 Department of Radiology, Tokushima Prefectural Central Hospital, Tokushima, Japan; 4 Department of Radiology, Tsurugi Municipal Handa Hospital, Tokushima, Japan; 5 Department of Community Medicine and Medical Science, Tokushima University Graduate School of Biomedical Sciences, Tokushima, Japan

**Keywords:** Pulmonary hypertension, Pulmonary hypertension classification, Ventilation–perfusion scintigraphy, Chronic thromboembolic pulmonary hypertension, Pulmonary arterial hypertension

## Abstract

Pulmonary hypertension encompasses a diverse set of conditions characterized by increased pressure in the pulmonary arteries. Proper classification is crucial for effective diagnosis and treatment. Ventilation–perfusion scintigraphy plays a vital role in imaging, especially in differentiating chronic thromboembolic pulmonary hypertension from other types of pulmonary hypertension. This article reviews the clinical applications of ventilation–perfusion scintigraphy in the classification of pulmonary hypertension, with particular focus on its relevance to groups 1 (pulmonary arterial hypertension), 3 (pulmonary hypertension associated with lung diseases or hypoxia), and 4 (chronic thromboembolic pulmonary hypertension). In addition, it explores recent technological advances and their impact on clinical practice.

## Introduction

Pulmonary hypertension (PH) is caused by a wide range of disorders and is defined by an elevated mean pulmonary arterial pressure (mPAP) greater than 20 mmHg at rest according to the 2022 European Society of Cardiology/European Respiratory Society (ESC/ERS) guidelines [1]. Japanese criteria define PH as an mPAP of 25 mmHg or more at rest [2]. PH is classified into five distinct groups based on its underlying cause, including: Group 1 (pulmonary arterial hypertension [PAH]), Group 2 (PH related to left heart disease), Group 3 (PH due to lung conditions or hypoxia), Group 4 [chronic thromboembolic PH (CTEPH)], and Group 5 (PH with complex or unclear mechanisms). Among these, ventilation–perfusion (V/Q) scintigraphy is particularly effective in diagnosing and categorizing CTEPH and is garnering attention for its role in evaluating other types of PH [3]. A normal V/Q scintigraphy can reliably rule out CTEPH, with a reported sensitivity between 96 and 97% and a specificity between 90 and 95% [4].

Single-photon emission computerized tomography (SPECT) and SPECT/computed tomography (CT) identify perfusion abnormalities that might be overlooked by planar scintigraphy, thereby avoiding underestimation of the extent of lesions. The ESC/ERS guidelines now recommend V/Q SPECT over planar imaging to evaluate PH [1]. V/Q SPECT offers greater diagnostic accuracy compared to planar scans, contributing to fewer inconclusive results [5, 6]. Nonetheless, a recent prospective investigation reported no statistically significant difference in diagnostic performance between V/Q SPECT and planar imaging in patients with CTEPH [7]. This outcome may be attributed to the presence of multiple large perfusion defects, which are typically visible on planar imaging, in most patients with CTEPH. However, at the segmental level, SPECT has demonstrated superior sensitivity when compared with planar scintigraphy for detecting perfusion abnormalities [7, 8]. This review summarizes the role of V/Q scintigraphy in classifying PH, with a particular focus on its diagnostic performance and expanding clinical utility.

## PH overview

PH is a multifaceted condition that presents with nonspecific symptoms such as shortness of breath during physical activity, fatigue, and chest discomfort, which can lead to delays in diagnosis [1]. If left untreated, PH can progress to right-sided heart failure and may be fatal. Classification of PH based on the underlying pathophysiology is the key to determining appropriate therapeutic interventions. The clinical classification of PH based on the ESC/ERS guidelines is summarized in Table 1. CTEPH is a rare consequence of acute pulmonary embolism [1, 9–14], resulting from residual clots that fail to dissolve completely. The clots organize over time, leading to the narrowing or blockage of the pulmonary arteries. This process increases pulmonary vascular resistance and pulmonary arterial pressure. In earlier cases, the standard approach if surgery was not feasible involved medical therapy using pulmonary vasodilators. However, in recent years, balloon pulmonary angioplasty (BPA) has become a viable alternative, now demonstrating treatment outcomes comparable to those of surgical pulmonary endarterectomy (PEA) [15–17].

**Table 1:** Clinical classification of pulmonary hypertension

Group 1. Pulmonary arterial hypertension
1.1 Idiopathic
1.1.1 Non-responders at vasoreactivity testing
1.1.2 Acute responders at vasoreactivity testing
1.2 Heritable
1.3 Associated with drugs and toxins
1.4 Associated with
1.4.1 Connective tissue disease
1.4.2 HIV^a^ infection
1.4.3 Portal hypertension
1.4.4 Congenital heart disease
1.4.5 Schistosomiasis
1.5 PAH^b^ with features of venous/capillary (PVOD^c^/PCH^d^) involvement
1.6 Persistent PH^e^ of the newborn
Group 2. PH associated with left heart disease
2.1 Heart failure
2.1.1 with preserved ejection fraction
2.1.2 with reduced or mildly reduced ejection fraction
2.2 Valvular heart disease
2.3 Congenital/acquired cardiovascular conditions leading to post-capillary PH
Group 3. PH associated with lung diseases and/or hypoxia
3.1 Obstructive lung disease or emphysema
3.2 Restrictive lung disease
3.3 Lung disease with mixed restrictive/obstructive pattern
3.4 Hypoventilation syndromes
3.5 Hypoxia without lung disease (e.g., high altitude)
3.6 Developmental lung disorders
Group 4. PH associated with pulmonary artery obstructions
4.1 Chronic thromboembolic PH
4.2 Other pulmonary artery obstructions
Group 5. PH with unclear and/or multifactorial mechanisms
5.1 Hematological disorders
5.2 Systemic disorders
5.3 Metabolic disorders
5.4 Chronic renal failure with or without hemodialysis
5.5 Pulmonary tumor thrombotic microangiopathy
5.6 Fibrosing mediastinitis

^1^Adapted from the 2022 ESC/ERS Guidelines

^a^HIV, human immunodeficiency virus

^b^PAH, pulmonary arterial hypertension

^c^PVOD, pulmonary veno-occlusive disease

^d^PCH, pulmonary capillary hemangiomatosis

^e^PH, pulmonary hypertension

## V/Q scintigraphy: principles and techniques

### Ventilation imaging

Lung ventilation can be assessed using radiolabeled aerosols, such as Technetium-99mTc-diethylenetriaminepentaacetic acid (^99m^Tc-DTPA), or Technegas® (Cyclopharm Ltd., Sydney, Australia), or by inhalation of noble gases, such as krypton-81m (^81m^Kr). The aerosolized form of ^99m^Tc-DTPA contains particles typically ranging from 1.2 to 2 μm in diameter and allows evaluation of alveolar-capillary membrane permeability [18]. Technegas, composed of ultrafine ^99m^Tc-labeled carbon particles (approximately 0.005–0.2 μm), behaves like a gas in the bronchial tree but settles in peripheral lung areas by diffusion [19]. It is recommended to use Technegas within 10 min of preparation to achieve optimal imaging quality due to aggregation over time [20]. Technegas is now approved for clinical use in Japan, although its availability may vary among institutions. ^81m^Kr, a radioactive gas with a short 13 s half-life, is inhaled continuously to maintain steady-state activity in the alveoli during image acquisition. Its higher photon energy (190 keV) compared with that of ^99m^Tc (140 keV) permits the simultaneous acquisition of V/Q scintigraphy, improving efficiency. SPECT acquisition is increasingly used for ventilation imaging, offering improved spatial resolution and quantification compared to planar imaging.

### Perfusion imaging

**Fig. 1:**
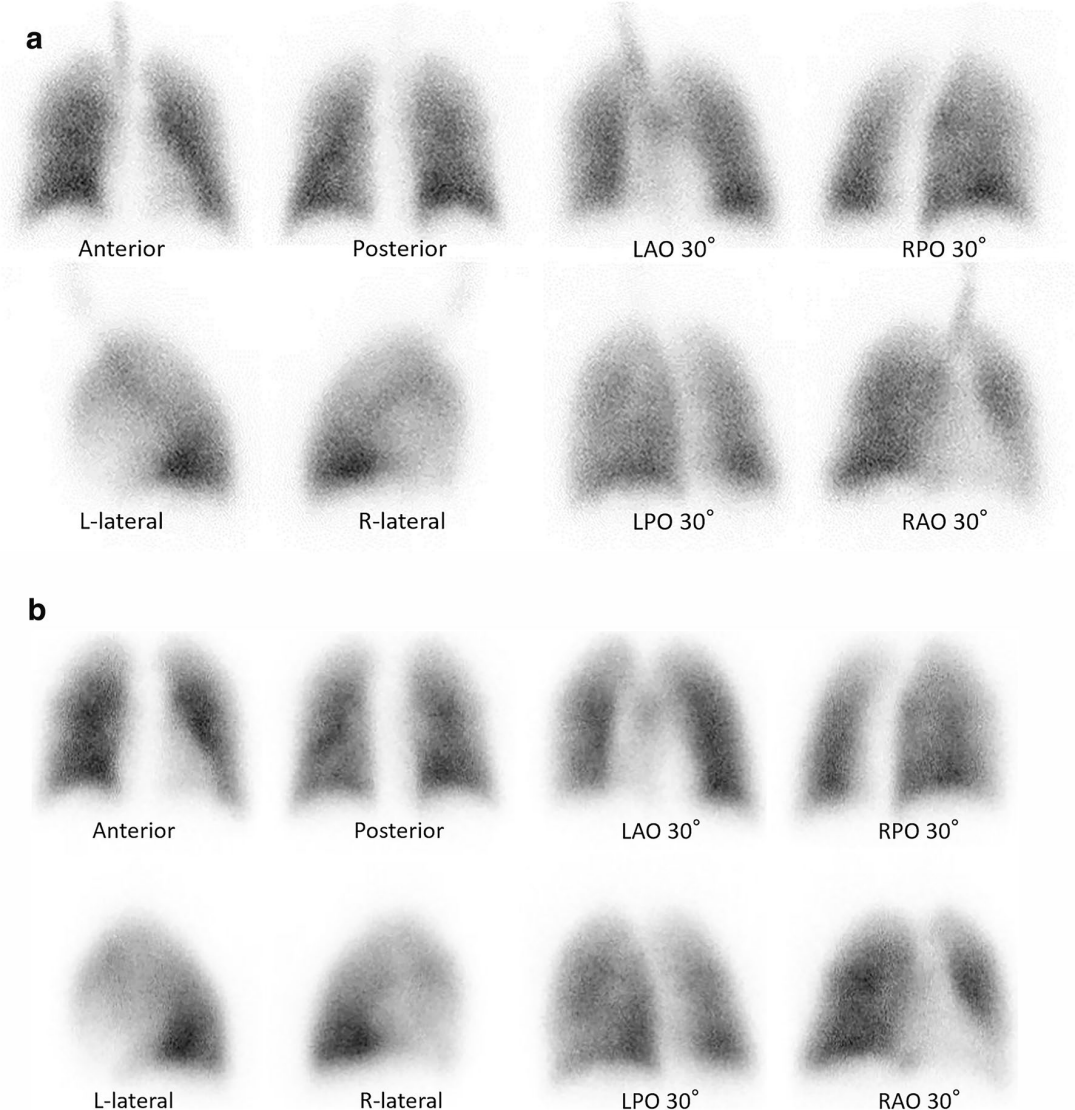
Normal ventilation and perfusion scintigraphy. **a** Ventilation scan using ⁸^1m^Kr^a^ Alternative agents include xenon-133 and Technegas^®^ (Cyclopharm Ltd., Sydney, Australia). After inhalation, ⁸^1m^Kr distributes evenly throughout the lungs and is exhaled, allowing evaluation of pulmonary ventilation. Due to its short physical half-life of 13 s, continuous inhalation is required. ⁸^1m^Kr is not suitable for washout phase imaging. A closed breathing circuit is not necessary. **b** Perfusion scan using ⁹⁹^m^Tc-MAA^b^. The particles are larger than pulmonary capillaries, allowing them to lodge as microemboli and reflect regional pulmonary blood flow. Imaging typically includes multiple planar chest views as well as SPECT^c^ or SPECT/CT^d^ acquisition. Because the particles tend to aggregate, backflow of blood into the syringe must be avoided. The tracer distribution is influenced by the patient’s posture at the time of injection. In pulmonary hypertension, elevated vascular pressures can lead to perivascular edema and result in reduced pulmonary perfusion. Anterior, posterior, lateral, and oblique views are shown to illustrate standard imaging orientations used in clinical interpretation. Oblique views are labeled in the images as RAO 30°, RPO 30°, LAO 30°, and LPO 30°, and lateral views as L-lateral and R-lateral, corresponding to standard clinical practice. ^a^⁸^1m^Kr, krypton-81m. ^b^⁹⁹^m^Tc-MAA, technetium-99m-labeled macroaggregated human serum albumin. ^c^SPECT, single-photon emission computed tomography. ^d^CT, computed tomography

Macroaggregated albumin particles labeled with ^99m^Tc are intravenously administered for perfusion imaging. These particles, measuring 15–100 μm, temporarily occlude pulmonary capillaries, enabling visualization of regional blood flow. Although a dose of 60,000 particles may be sufficient, approximately 400,000 particles are commonly used to ensure image quality while maintaining minimal vascular obstruction [21]. To prevent artifacts, it is essential to avoid the backflow of blood into the syringe, which could lead to particle clumping and result in false hotspots on the scan. A 21-gauge or larger needle is generally recommended, and the injection should be performed in the sitting position to minimize artifact due to uneven distribution. In addition to planar imaging, perfusion SPECT or SPECT/CT is increasingly utilized to better visualize segmental perfusion defects. Figure 1 presents representative images of normal V/Q scintigraphy to illustrate the standard physiological distribution.

## Clinical applications in PH classification

V/Q scintigraphy is a valuable diagnostic tool for the assessment of PH, especially for differentiating CTEPH from other PH subtypes. When a radiotracer is administered intravenously to healthy individuals in a sitting position, gravity causes the tracer to accumulate predominantly in the lower lung fields. However, in patients with PH, this distribution often shifts toward the upper lung zones because of vascular remodeling and elevated pulmonary pressure [22]. A normal perfusion scan strongly suggests the absence of CTEPH [23], thus distinguishing CTEPH from other causes of PH. It remains the imaging modality of choice for the initial screening of chronic thromboembolic diseases. Although SPECT is more sensitive than planar imaging for detecting perfusion defects, both techniques are appropriate for the initial assessment.

According to guidelines from the ESC, V/Q scintigraphy is the recommended initial imaging test for evaluating patients with suspected CTEPH following an episode of acute pulmonary embolism [24]. Planar scans are a reliable first-line option, providing excellent sensitivity (96–97%) and specificity (90–95%) for diagnosing CTEPH [4]. Although SPECT shows slightly lower sensitivity at the segmental artery level, it is highly effective in detecting clinically significant disease [24].

The 2019 European Association of Nuclear Medicine guidelines for V/Q scintigraphy do not distinguish acute pulmonary embolism from chronic thromboembolic disease [9]. In patients with CTEPH, perfusion scintigraphy often reveals segmental or subsegmental defects, while ventilation images remain largely normal, resulting in the characteristic mismatched pattern [7, 9, 25]. This mismatch is a hallmark of Group 4 PH, providing strong diagnostic evidence for CTEPH and related embolic conditions.

### Group 1: PAH

In Group 1 PAH, lung perfusion scintigraphy often appears normal or reveals scattered, non-segmental perfusion defects, commonly described as a “mottled” pattern [26]. Some patients demonstrate nearly homogeneous perfusion, whereas others present with diffuse or patchy abnormalities [23], distinguishing them from the segmental or lobar defects observed in thromboembolic diseases such as CTEPH [27–29].

According to Hayashida et al., in patients with early stages of PAH, the lung parenchyma remains intact despite elevated pulmonary pressure. However, as the disease progresses, parenchymal injury may become widespread, resulting in the mottled appearance across the lung fields [30]. Importantly, the patchy or “moth-eaten” perfusion defects typical of PAH can resemble those observed in CTEPH, which may lead to an incorrect diagnosis of thromboembolic disease [8].

### Group 2: PH due to left heart disease

Group 2 PH results from left heart disease such as heart failure or valvular disorders [23]. In this group, V/Q scintigraphy typically shows no characteristic segmental perfusion defects. Perfusion may appear relatively homogeneous, indicating a lack of segmental defects rather than the absence of gravity-dependent distribution. In advanced cases, diffuse perfusion abnormalities associated with pulmonary congestion and interstitial edema may be seen. Unlike chronic thromboembolic pulmonary hypertension (CTEPH), segmental mismatched perfusion defects are uncommon. Differentiating Group 2 PH from CTEPH is important, particularly in patients with overlapping risk factors, and requires integration of clinical, hemodynamic, and imaging data.

### Group 3: PH due to lung diseases and/or hypoxia

Group 3 PH, associated with lung diseases and/or hypoxia, is common in patients with advanced parenchymal and interstitial lung disease [1]. A heterogeneous ventilation pattern is a characteristic of chronic obstructive pulmonary disease (COPD) observed on V/Q SPECT/CT [31]. In V/Q SPECT images, patients with COPD demonstrate matched V/Q defects and/or reversed V/Q mismatches [32]. Since the extent of matched defects increases with an increasing degree of emphysema, V/Q SPECT is useful not only for diagnosing COPD, but also for assessing its severity [33]. In cases of interstitial pneumonia, areas of V/Q mismatch with normal ventilation and hypoperfusion are mixed with areas of matching V/Q defects in the honeycomb region [34, 35]. The significance of using scintigraphy to evaluate PH from chronic lung disease has yet to be established. However, noninvasive assessment of the percentage of pulmonary perfusion defects appears to be a valuable tool for evaluating the severity of pulmonary circulation impairment [36].

### Group 4: CTEPH

V/Q scintigraphy is the gold-standard screening tool for CTEPH [1, 3]. Characteristic findings include segmental or larger perfusion defects with preserved ventilation, resulting in a classic mismatch pattern. This hallmark pattern is vital for distinguishing CTEPH from other types of PH and guides decisions about further diagnostic imaging and treatment [3, 4]. However, these findings do not always indicate CTEPH [37, 38]. Several other conditions may present with similar scintigraphic findings including in situ thrombosis, pulmonary artery sarcoma, fibrosing mediastinitis, pulmonary vasculitis, pulmonary vein stenosis secondary to atrial fibrillation ablation, sarcoidosis, and other rare conditions that mimic CTEPH on imaging. As CTEPH often affects multiple vascular territories, perfusion defects can be extensive. Planar and SPECT imaging provide adequate sensitivity, although SPECT offers more precise localization [1, 5, 6]. The key to an accurate diagnosis is recognizing large mismatched perfusion defects that are not explained by parenchymal disease.

**Fig. 2:**
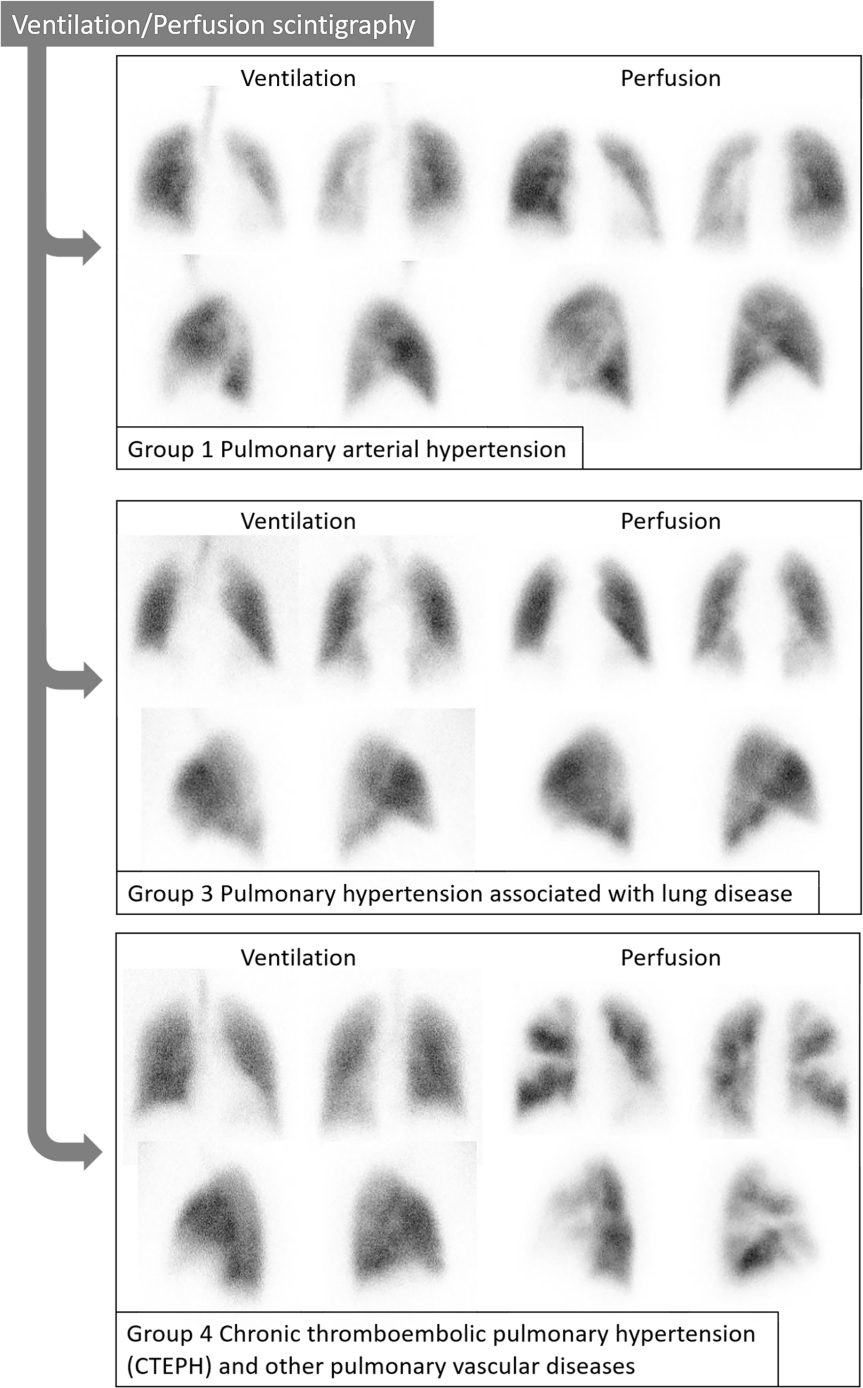
Representative V/Q^a^ scintigraphy images of patients with PH^b^ caused by mechanisms from Groups 1, 3, and 4. Each panel shows planar V/Q images consisting of anterior and posterior views in the upper rows and lateral views in the lower rows. Group 1 (pulmonary arterial hypertension) demonstrates relatively homogeneous perfusion without segmental defects. Group 3 (PH due to lung diseases) shows patchy perfusion defects corresponding to underlying parenchymal abnormalities. Group 4 (chronic thromboembolic pulmonary hypertension) reveals mismatched segmental perfusion defects characteristic of chronic thromboembolic disease. ^a^V/Q, ventilation/perfusion. ^b^PH, pulmonary hypertension

Figure 2 shows representative V/Q scintigraphy images of patients with PH representing Groups 1, 3, and 4. Table 2 summarizes the characteristic imaging findings on V/Q scintigraphy and contrast-enhanced CT in these groups.

**Table 2:** Comparison of imaging findings in pulmonary hypertension classification groups

Imaging modality	Group 1: PAH^a^	Group 3: PH^b^ associated with lung disease	Group 4: CTEPH^c^ and other pulmonary vascular diseases
Contrast-enhanced CT^d^	No thrombi	No thrombi; parenchymal findings	Thrombi or vascular abnormalities
V/Q^e^ scintigraphy	Normal or mottled perfusion defects	Matched perfusion and ventilation abnormalities	Segmental perfusion defects without ventilation abnormalities

Group 2 (PH due to left heart disease) typically shows no characteristic perfusion defects on V/Q scintigraphy and is therefore not depicted in this table

^a^PAH, pulmonary arterial hypertension

^b^PH, pulmonary hypertension

^c^CTEPH, chronic thromboembolic pulmonary hypertension

^d^CT, computed tomography

^e^V/Q, ventilation/perfusion

**Fig. 3:**
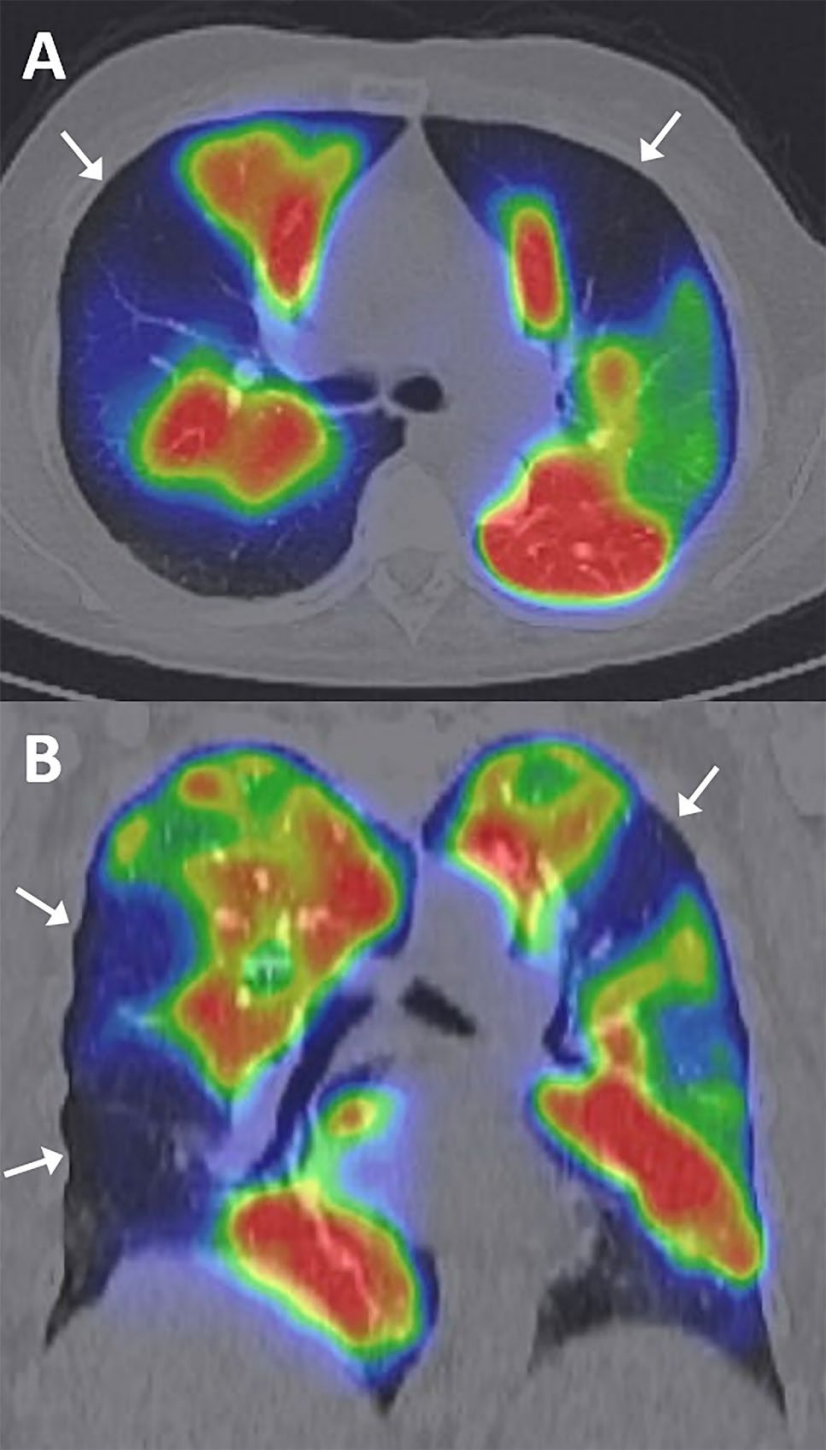
Representative perfusion SPECT/CT fusion images of a patient with chronic thromboembolic pulmonary hypertension (CTEPH). Axial (**A**) and coronal (**B**) views demonstrate multiple segmental perfusion defects, particularly in the peripheral regions of both lungs (white arrows). These findings are characteristic of CTEPH. Ventilation SPECT imaging was not available at our institution

In addition, Fig. 3 presents a perfusion SPECT image from a patient with CTEPH, demonstrating segmental perfusion defects with high anatomical clarity. Although ventilation SPECT was not acquired at our institution, perfusion SPECT alone provided clinically valuable diagnostic information.

## V/Q scintigraphy vs. other modalities

CT pulmonary angiography (CTPA) is widely accessible and commonly available 24 h a day. It helps to identify alternative causes of PH, such as pulmonary fibrosis, emphysema, fibrosing mediastinitis, and malignancy, thereby narrowing the differential diagnosis [39]. However, compared with V/Q scintigraphy, CTPA has certain diagnostic and technical limitations, including the use of iodinated contrast agents and reports of technically suboptimal scans in up to 11% of cases [40]. Although CTPA is suitable for detecting proximal CTEPH, it may fail to identify distal diseases, even when the image quality is high, thereby limiting its ability to exclude CTEPH [41].

Dual-energy CT (DECT) enables the assessment of pulmonary perfusion through the reconstruction of iodine-based perfusion maps, offering improved visualization in cases of distal CTEPH [39]. The perfusion patterns in patients with CTEPH observed with DECT differ markedly from images observed in those with PAH [42], and DECT can help distinguish acute pulmonary embolism from chronic thromboembolic disease by evaluating the attenuation of the embolic material [43]. Quantification of the pulmonary perfused blood volume (PBV) using DECT is an objective method for assessing disease severity [44, 45]. Several small studies have demonstrated modest to strong correlation between PBV maps and findings on planar imaging or V/Q SPECT [42, 46, 47]. Additional benefits of DECT include high diagnostic accuracy, low radiation exposure, and the ability to simultaneously evaluate cardiovascular and pulmonary anatomy, including the pulmonary arteries, heart chambers, shunt vessels, and lung parenchyma. Lung abnormalities such as emphysema, interstitial lung disease, inflammation, and tumors can be identified [48]. Moreover, DECT provides rapid image acquisition and reconstruction, typically completed in less than 5 min, whereas V/Q SPECT may require 25–30 min depending on the protocol [48].

Perfusion MRI has also shown excellent diagnostic accuracy for CTEPH, with a reported sensitivity of 97%, specificity of 92%, positive predictive value of 95%, and negative predictive value of 96%, which are comparable to those of V/Q scintigraphy and CTPA [49]. In a study comparing contrast-enhanced MR pulmonary angiography (CE-MRA), CTPA, and digital subtraction angiography (DSA), CE-MRA achieved a sensitivity and specificity of 83.1% and 98.6%, respectively, at the main/lobar level and 87.7% and 98.1%, respectively, at the segmental level. However, DSA was superior in identifying subsegmental artery involvement [50].

Although MRI provides a noninvasive option for diagnosing and monitoring CTEPH, its clinical adoption remains limited owing to inconsistent availability, longer scan times, and the need for specialized expertise in image interpretation.

## Advances and future directions

Recent advances in imaging technology, particularly the development of hybrid SPECT/CT, have contributed to the improved diagnostic accuracy of V/Q scintigraphy. Looking ahead, the integration of artificial intelligence (AI) for automated image interpretation, as well as the integration of V/Q scintigraphy with complementary modalities such as cardiac MRI and PET/CT, holds promise for further enhancement of diagnostic precision. PEA remains the first-line treatment for operable cases of CTEPH, and BPA has emerged as a valuable therapeutic option. The most recent guidelines from the ESC/ERS recommend BPA as a Class I treatment for patients with inoperable disease [1, 51]. Changes in perfusion defect scores after BPA may serve as useful imaging biomarkers for monitoring treatment responses and evaluating improvements in pulmonary hemodynamics in patients with CTEPH [52]. The application of AI-based tools such as Auto Lung 3D (Siemens Healthineers, Knoxville, USA), which automatically segments lung lobes based on CT data acquired during SPECT imaging, represents a significant advancement in imaging analyses [53]. Automated quantification of lobar tracer uptake using such tools may improve both diagnostic accuracy and workflow efficiency.

## Conclusion

V/Q scintigraphy is the foundational imaging modality in the diagnosis and classification of PH, particularly CTEPH. As imaging technologies continue to evolve, the role of V/Q scintigraphy in the assessment of other subtypes of PH has expanded. The future of V/Q scintigraphy lies in the adoption of hybrid systems, automation, and AI-driven tools that enhance the diagnostic accuracy, streamline workflows, and provide more objective assessments. These advances will likely contribute to improved clinical outcomes through earlier and more precise diagnoses and better monitoring of treatment efficacy.
